# Circadian Clock and Complement Immune System—Complementary Control of Physiology and Pathology?

**DOI:** 10.3389/fcimb.2020.00418

**Published:** 2020-08-14

**Authors:** Pooja Shivshankar, Baharan Fekry, Kristin Eckel-Mahan, Rick A. Wetsel

**Affiliations:** ^1^Research Center for Immunology and Autoimmune Diseases, Brown Foundation Institute of Molecular Medicine, McGovern Medical School, University of Texas Health Science Center at Houston, Houston, TX, United States; ^2^Center for Metabolic and Degenerative Diseases, McGovern Medical School, University of Texas Health Science Center at Houston, Houston, TX, United States

**Keywords:** autoimmune disease, inflammation immunomodulation, metabolic disease, circadian system, complement immunity, host microbial interactions

## Abstract

Mammalian species contain an internal circadian (i.e., 24-h) clock that is synchronized to the day and night cycles. Large epidemiological studies, which are supported by carefully controlled studies in numerous species, support the idea that chronic disruption of our circadian cycles results in a number of health issues, including obesity and diabetes, defective immune response, and cancer. Here we focus specifically on the role of the complement immune system and its relationship to the internal circadian clock system. While still an incompletely understood area, there is evidence that dysregulated proinflammatory cytokines, complement factors, and oxidative stress can be induced by circadian disruption and that these may feed back into the oscillator at the level of circadian gene regulation. Such a feedback cycle may contribute to impaired host immune response against pathogenic insults. The complement immune system including its activated anaphylatoxins, C3a and C5a, not only facilitate innate and adaptive immune response in chemotaxis and phagocytosis, but they can also amplify chronic inflammation in the host organism. Consequent development of autoimmune disorders, and metabolic diseases associated with additional environmental insults that activate complement can in severe cases, lead to accelerated tissue dysfunction, fibrosis, and ultimately organ failure. Because several promising complement-targeted therapeutics to block uncontrolled complement activation and treat autoimmune diseases are in various phases of clinical trials, understanding fully the circadian properties of the complement system, and the reciprocal regulation by these two systems could greatly improve patient treatment in the long term.

## Introduction

Circadian rhythms, oscillations of ~24-h periodicity, are critical for a healthy immune system. Both innate and adaptive immunity are regulated by the circadian clock at the level of the circadian pacemaker, the suprachiasmatic network (SCN), as well as by peripheral clocks in other tissues (Laste et al., 2013; Perfilyeva et al., 2017). Circadian rhythms are synchronized to the light/dark cycle of our environment via the SCN of the hypothalamus, which responds directly to light information transmitted through the retinal ganglion cells (Ruby et al., 2002; Lucas et al., 2014). The pacemaker conveys information to so called “peripheral clocks” through indirect and direct mechanisms (Welsh et al., 2010), ultimately controlling rhythmicity in the sleep/wake cycle and energy intake and metabolism. Prior to electricity, humans confined their sleep/wake cycles more closely to the rising and the setting of the sun. However, due in part to the urbanized lifestyles and more flexible working hours, adherence to a rigid 24-hr. schedule is often compromised, resulting in sleep deprivation and circadian disruption in the form of “social jet-lag” (Brisbare-Roch et al., 2007; Yao et al., 2015; Pagel et al., 2017; Gupta, 2019; Walker et al., 2020). There is evidence that circadian disruption may alter the proper temporal regulation of proinflammatory cytokines, which can induce uncontrolled complement activation, resulting in neuropathological disorders, autoimmune diseases, and shortened life expectancy (Petersen et al., 1986; Ricklin, 2012; Gomez-Gonzalez et al., 2013; Hurtado-Alvarado et al., 2013; Brodsky, 2015; Oster et al., 2017; Jasim et al., 2019; Inokawa et al., 2020).

**Figure 1:**
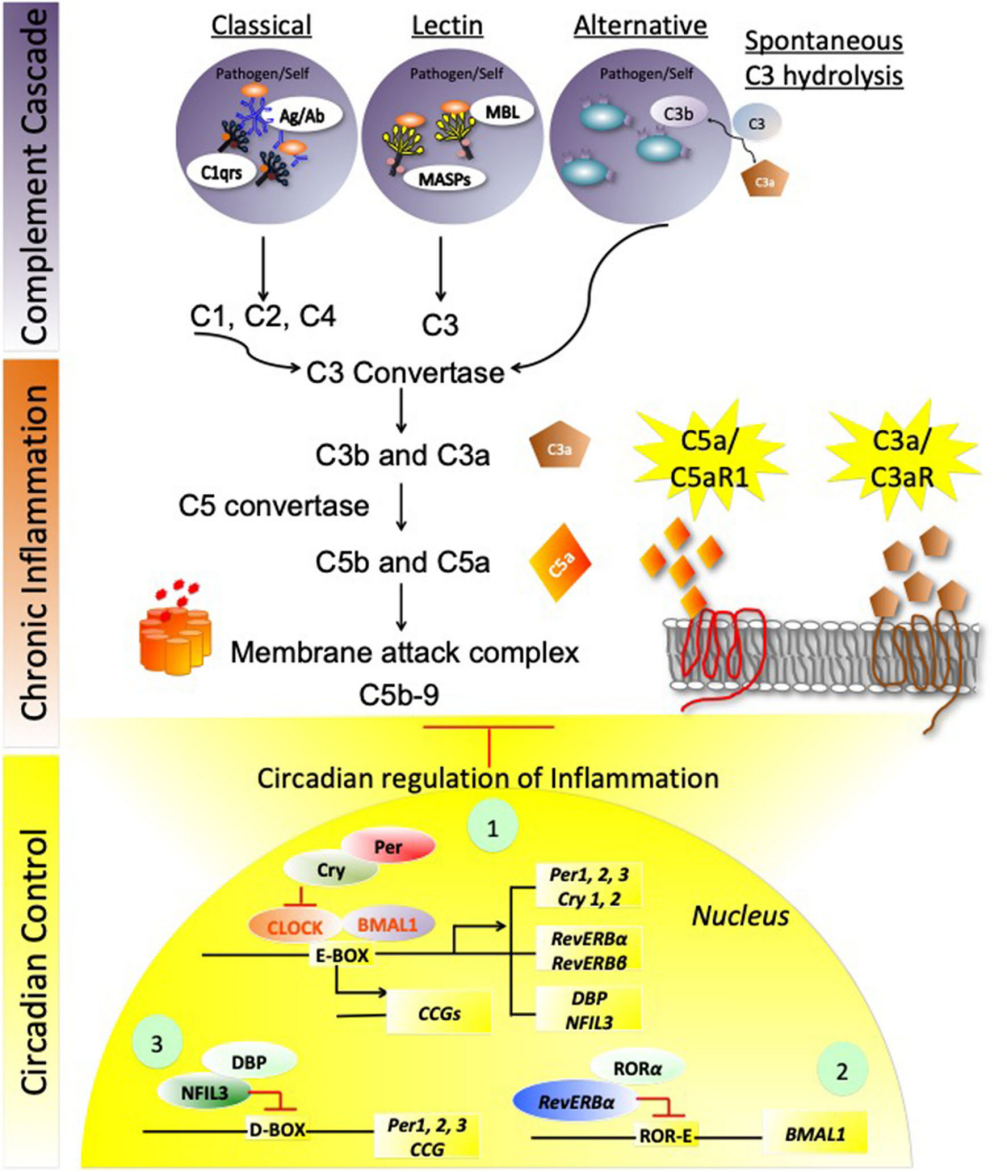
Circadian regulation of complement immune system. The stepwise schema of three different Complement cascade pathways originating from three different stimuli is depicted in the top panel. (1) The classical pathway is being activated by antigen (from pathogens or self-antigens) and antibody (IgM and IgG) interaction, leading to activation of C3. (2) The Lectin pathway recognizes pathogen-associated carbohydrate moieties via lectins, and leading to activation of C3, by classical C3 convertase. (3) The alternate complement pathway is activated through spontaneous hydrolysis of C3 and binding of C3b to the pathogens or self-antigens directly to further activate C3, by C3 convertase. C3 activation to generate C5 convertase, thereby activating Membrane attack complex (MAC) formation. C3a and C5a are the two anaphylatoxins that signal through their respective receptors, C3aR and C5aR1 on myeloid and non-myeloid cells, and enhance inflammation and chemotaxis. These inflammatory pathways are tightly regulated under normal physiological conditions by circadian Clock:BMAL1 regulated signaling, that involves three circuits: The first circuit involves the transcriptional activation of Clock-BMAL1 control proteins, *Per1-3/Cry1-2, Rev-erb*α*/*β, and *DBP/NFIL-3*. Cry and Per regulate Clock: BMAL1 expression by binding to the Clock subunit at the E-box enhancer elements and repressing Clock: BMAL1-mediated transcriptional activation of several *Circadian controlled genes* (*CCGs*), besides the three circadian loops. The second circuit of repression involves Rev-ERBα along with RORα (RAR-related orphan receptor alpha (RORα), repressing *BMAL1* by the ROR/REV-ERB-response element (RORE)-dependent mechanism. The third circuit employs NFIL-3 and DBP-mediated repression of *Per 1,2,3* along with several proinflammatory mediators' expression, and CCGs, thereby controlling complement activation and exacerbated inflammation. Explanations of these pathways are detailed in the text.

Complement activation is an integral process involved in host innate and adaptive immune responses. Distinct stimuli activate three different complement pathways: these include (1) classical, (2) lectin, and (3) alternate pathways. The classical and the lectin pathways converge with the alternate complement pathway resulting in modulation of host innate as well as adaptive immunity and opsonization (destruction) of pathogens (Arkhipova et al., 2012; Verschoor and Langer, 2013; Brodsky, 2015; Cedzynski et al., 2019). The organization of the three different Complement pathways originating from three different stimuli is depicted in Figure 1 (top). The classical pathway is activated by antigen (from pathogens or self-antigens) and antibody (IgM and IgG) interactions. The antigen/antibody complex is recognized by C1q, which is complexed with other two factors C1r, and C1s. This interaction subsequently activates C2 and C4, leading to activation of C3, by classical C3 convertase (composed of fragments of C4 and C2, the C4b and C2b proteins) (Ding et al., 2013). The lectin pathway recognizes pathogen-associated carbohydrate moieties, such as mannose sugars via interaction with mannose binding lectins, thereby activating MBL-associated serine proteases (MASPs). These MASPs further activate C3, by classical C3 convertase (Fujita, 2002; Fujita et al., 2004). The alternate complement pathway is activated through spontaneous hydrolysis of C3 and binding of C3b to the pathogens or self-antigens directly, to further activate C3 by C3 convertase (fragments of C3: C3b, factor Bb). All the three cascades converge at C3 activation to generate C5 convertase (C3b, factor Bb-C3b complex), resulting in Membrane Attack Complex formation (C5b-9: C5b, C6, C7, C8, and polymeric C9). C3a and C5a peptides are potent cytokines that signal through their respective receptors, C3aR and C5aR1 on myeloid and non-myeloid cells, thereby increasing inflammation and chemotaxis (Markiewski and Lambris, 2007; Dunkelberger and Song, 2010; Holers, 2014). Although the alternate complement pathway acts as a surveillance mechanism in healthy tissues, dysregulation of the alternate complement pathway can result in chronic inflammatory conditions including, but not limited to, inflammatory bowel diseases (IBD), cardiac inflammation, autoimmune diseases, and glomerulonephropathies (Aiyaz et al., 2012; Carter, 2012; Fearn and Sheerin, 2015; Wadhwa et al., 2019). These conditions usually arise in part from uncontrolled production of complement anaphylatoxins of the alternate complement pathway. The complement factors include C3a and C5a, which signal via their specific target receptors C3aR (Ratajczak et al., 2004; Wende et al., 2013; Mueller-Ortiz et al., 2014; Muenstermann et al., 2019) and C5aR1 (Haviland et al., 1995; Wetsel, 1995). Complement receptors are members of the G-protein coupled seven transmembrane receptors (Hodgson et al., 1977; Fujiwara et al., 1981; Richens et al., 1982; Gasque et al., 2000; Alexander et al., 2006). Within physiological concentrations, both C3a and C5a are highly reactive peptides and are subject to carboxypeptidase N-mediated degradation to C3a-desArg and C5a-desArg. These complement conjugates are less compatible with their respective receptors, therefore unable to fully control inflammation and chemotaxis. The C4a anaphylatoxin generated by complement C4 in the complement cascade is the least potent anaphylatoxin and is not known to effectively induce inflammation or chemotaxis (Wild et al., 1990). This is due in part to the fact that it is very rapidly degraded by the carboxypeptidases into the inactive form of C4a-desArg.

Of all the three anaphylatoxins C5a is the most potent with ~2,500-fold more potency than C4a, and 50-fold more potent than C3a (Gorski et al., 1979; Mak and Saunders, 2006; Barnum, 2015). In addition to the complement factors, complement regulatory factors, such as factor H, C1q, and decay accelerating factor (DAF) recognize both self and non-self-inflammatory cues (Kawano, 2000), suppressing inflammation under normal physiological conditions (Brodsky, 2015).

Studies have demonstrated an interaction of innate and adaptive immune responses in lymphocytic differentiation, skewing, polarization and activation of B and T lymphocytes in rheumatoid arthritis (RA), systemic lupus erythromatosis (Gibbs et al., 2012), and multiple sclerosis (Youinou et al., 1984; Kumagai et al., 1989; Sakane et al., 1991; Berek and Kim, 1997; Buntinx et al., 2002; Blaschke et al., 2003; De Miguel et al., 2003; Li et al., 2006a,b; Chiang et al., 2011; Moura et al., 2011; Caporali et al., 2014; Zhang et al., 2015). However, during uncontrolled complement activation, much of the impact of complement peptides C3a/C3aR and C5a/C5aR1 is exerted upon adaptive immune effector CD4^+^ T-cells. Specifically, overactivation of complement factors leads to CD4^+^ T cell polarization to Th1, Th2, and Th17 resulting in exacerbation of inflammation and pathophysiological consequences (Fang et al., 2009; Shivshankar et al., 2020).

Though the circadian clock system is an endogenous time keeping system that is active in almost all cells of the body, regulating diverse processes, such as sleep, metabolism, and synaptic plasticity, surprisingly little is known about the circadian clock in complement factor regulation. However, there is evidence suggestive of crosstalk between these regulatory systems in controlling immune response (Figure 1 bottom) (Kim et al., 1980; Reis et al., 2011). In this review, we will focus on (1) how complement and sleep affects host immunosurveillance, (2) how complement and circadian signaling are altered in immunopathogenesis and autoimmune conditions, and (3) how oxidative stress and altered complement signaling affect circadian action in metabolic disorders. Figure 1 summarizes the three complement cascade pathways that are reportedly involved in chronic inflammation, which evidence suggests may be perturbed by circadian dysregulation.

## Co-Regulatory Roles of Complement and Sleep on Immunosurveillance

Immunosurveillance is maintained by the constant flow of hematopoietic stem cells (HPSCs) from the bone marrow and recirculation in the blood and lymph via a bioactive phosphoshingolipid, Spingosine-1 phosphate (S1P), which acts as a chemoattractant of HPSCs (Massberg et al., 2007). The number of HPSCs has been shown to be highly circadian, with the majority of HPSCs circulating in peripheral blood in the early morning hours (Golan et al., 2018; Adamiak et al., 2020; Ratajczak et al., 2020). Both S1P levels and the activated C5 complement pathway are reportedly circadian and play a role in the diurnal chemotaxis of HPSC egression of stem cells from bone marrow to the peripheral blood circulation to maintain immunosurveillance. In Budkowska et al. (2018), human peripheral blood was shown to have significantly increased complement activation markers, including complement anaphylatoxins' stable byproducts, C3adesArg and C5adesArg and the membrane attacking complex, MAC at 2.00 a.m. This complement activation preceded blood levels of S1P in the same volunteers, the latter of which reached a peak at 8.00 a.m. Using a mouse model devoid of C5 complement signaling, rhythmic HPSC recruitment could be entirely blocked, implicating the importance of circadian complement pathway activation in immune surveillance (Massberg et al., 2007; Janowska-Wieczorek et al., 2012; Ratajczak et al., 2012; Budkowska et al., 2018). While this study implicates rhythmic complement activation in the context of circulating HPSCs, other studies have shown a sleep-dependent increases in C3, C4, C3a, C5a, and complement factor I (CFI) ultimately resulting in dysregulated cytokines production (Reis et al., 2011; Manzar et al., 2016; Horvath et al., 2018; Wadhwa et al., 2019). Using the Pittsburgh Sleep Quality Index (PSQI) in a relatively small number of male university students, Manzar et al. demonstrated that poor sleep quality correlates with decreased proinflammatory C3 and C4 in the serum and increased levels of anti-inflammatory CFI during early bedtime and daytime (Manzar et al., 2015), suggesting that regular sleep patterns may be important for the normal cycling of complement factors. Supporting this link, increased serum C3a levels in both evening and daytime was correlated with severity of obstructive sleep apnea (Horvath et al., 2018). Under optimal physiological conditions, complement regulatory factors including CR1, membrane cofactor protein (MCP; also known as CD46), and Factor H control the complement activation at the levels of C3 convertase and inhibit the formation of C5-C9 MAC that could be detrimental to host cells. In particular factor H interacts with C3b on the host cell membranes and destabilizes C3 convertase and facilitates cleavage of C3b to inactive C3b (iC3b) and C3dg fragments; C3dg further gets cleaved into C3d, which helps in lowering the levels of circulating immune complexes (CIC), thereby limiting not only alternate complement pathway activation but also keeping chronic inflammation in control (Petersen et al., 1985, 1986; Dorval et al., 1989; Kashyap et al., 1992; Sanchez-Cuenca, 1994). Autoimmune conditions, such as SLE tend to dysregulate the balance of degradation of immune complexes which results in decreased C3d-mediated complex releasing activity and therefore increased levels of CICs (Sakurai et al., 1982). Interestingly, increased levels of CICs have been detected in blood collected during the daytime as compared to the nighttime from patients with active rheumatoid arthritis (Mageed et al., 1991a,b). In addition, the levels of C3 split product, C3d, the regulatory factor to reduce the burden of immune complexes in circulation, were significantly higher in the morning, with matching high pain scores in the morning. These data suggest that it is crucial to take into account the circadian and diurnal phases of complement activation in relevance to the pathogenesis and the immune cell responses (Petersen et al., 1986). In a study designed to assess the effects of the sleep/wake cycle on immunoregulatory properties of the complement, blood and blood-derived monocytes were analyzed under conditions of forced wakefulness (Reis et al., 2011). Interestingly, sleep deprivation resulted in a decrease in C3 activation (as measured by serum C3a levels). In addition, peripheral blood monocytes isolated from individuals undergoing sleep deprivation revealed significantly low levels of IL-12p70, when challenged with endotoxin lipopolysaccharide (LPS), suggesting that a sleep-dependent increase in complement factors may be important for normal immunosurveillance. Monocyte cultures isolated from these patients responded to LPS challenge in the presence of C5a with increased TLR4-driven IL12p70 production in the daytime harvested samples (10:00 a.m.), whereas monocytes collected from patients during the night (1:30 a.m.) did not respond to LPS challenge, suggesting a diurnal C5a/C5aR1 signaling axis the has physiologically distinct consequences based on the time of activation by complement factors (Reis et al., 2011).

## Mechanistic Connections Between Compliment and the Clock Derived From Pre-Clinical Studies

Though most laboratory mice are nocturnal, studies from mice also reveal a bidirectional relationship between immunity and the circadian clock. For example, mice challenged with the parasite *Leishmania major* showed a diurnally-dependent infection rate, with the number of neutrophils and macrophages infiltrating to the infection site variant by time of day (Kiessling et al., 2017). This variance is likely based on rhythmic expression of neutrophil and macrophage-attracting chemokines (Oghumu et al., 2010). In mice in which there is no functioning circadian clock in macrophages (Keller et al., 2009; Labrecque and Cermakian, 2015), rhythmicity in infection was eliminated (Kiessling et al., 2017). Thus, these data support a scenario where the host macrophage circadian clock appears to be necessary for rhythmicity of infectivity by *Leishmania major in vivo*.

Interactions between the clock and immune factors have even been noted at the level of the circadian pacemaker. For example, chronic low doses of LPS can alter the phase of the central pacemaker, a process mediated by the Toll-like receptor 4 (TLR4), a toll-like receptor belonging to the pattern recognition receptor family (Paladino et al., 2010). In addition, several studies have demonstrated that the time period from ZT9 to ZT15, which spans the ZT12 transition into the dark/active phase, differentially provokes immune cell response including polymorphonuclear cells (PMN) infiltration (Scheiermann et al., 2012), chemotaxis (Gibbs et al., 2012), enhanced bacterial clearance, and Toll-like receptor (TLR) activation for mounting inflammation and checkpoint controls (Muniain et al., 1988; Silver et al., 2012; Bellet et al., 2013; Pick et al., 2019). Using our own C3aR and C5aR1 deficient mice, we have demonstrated that C3aR plays a protective role against the LM challenge with mitigated inflammatory response and LM-induced apoptosis (Mueller-Ortiz et al., 2014). In addition, we have demonstrated the protective effects of C5aR1 signaling against LM infection to be mediated by Type-1 interferon signaling pathway (Calame et al., 2014). Using neutralizing type-1 interferon receptor prior to LM challenge *in vivo* in C5aR1 KO mice, we could rescue these mice from LM infection-induced mortality (Calame et al., 2014). Given that both C3aR and C5aR1 have been demonstrated to be pro-inflammatory as well as anti-inflammatory with regard to specific tissue pathology and infections (Klos et al., 2009), and the reported circadian regulation of C5 itself, it would be interesting to determine whether C5aR1-mediated protection to LM challenge is time-of-day dependent.

Besides C3aR and C5aR1, mammalian cells also express an additional complement decoy receptor, C5aR2 (also known as C5L2), which can sequester increased C5a and inhibit the C5aR1 signaling axis (Scola et al., 2009). In contrast to C3aR KO and C5aR1 KO mouse strains, C5aR2 KO mice have resistance toward systemic LM infections compared to their wildtype counterparts. This protection of C5aR2KO mice to LM challenge has been attributed to increased T cell responses, as these mice show significantly higher IL12p70 and IFN-γ production in splenic immune cells, which protects from systemic LM infection (Mueller-Ortiz et al., 2019). Though links between the decoy receptor itself and the circadian clock have not been established, based on its ability to sequester C5a, it too may have time-of-day consequences on immune response.

Several mouse models in which the circadian clock was either environmentally perturbed, or alternatively genetically manipulated have shown specific links between the circadian clock system and complement signaling. For example, in inbred mice exposed to alternate photoperiods, an increase in circulating B cells was noted in mice with short compared to long photoperiod, while long days produced an increase in the number of activated T cells (Pick et al., 2019). Interestingly, natural killer (Mueller-Ortiz et al., 2019) cell activity was increased under conditions of both alterations in photoperiod, suggesting immunological adaptation to circadian timing in some organisms (Yellon and Tran, 2002). These data are consistent with earlier studies performed on healthy women with sufficient nocturnal rest in which robust rhythms in the activity of natural killer (Mueller-Ortiz et al., 2019) cells was noted, with a peak in the morning or early afternoon (Gatti et al., 1987). Other studies have shown an interesting relationship between the clock system and complement at the level of the repressor protein PERIOD2, PER2. Specifically, PER2, through its negative regulation of the Circadian Locomotor Output Cycles Caps (CLOCK) aryl hydrocarbon receptor nuclear translocator like (ARNTL, or BMAL1) (BMAL:CLOCK) transcriptional complex, plays an indirect role in dampening natural killer cell activity against LPS challenge (Arjona and Sarkar, 2006; Liu et al., 2006; Sasaki et al., 2009), which could also be attributed to LPS- induced complement activation (Perlik et al., 2005). These studies corroborated with studies using mice with lesions of the central pacemaker, the SCN. Mice with lesioned SCN show resistance to LPS accompanied by a dampened innate immune response, and impaired energy metabolism (Coomans et al., 2013; Moravcova et al., 2018). Thus, both genetic and environmental disruption of the circadian clock system reveals previously unappreciated links between the circadian and complement systems.

## Complement and Circadian Signaling

**Figure 2:**
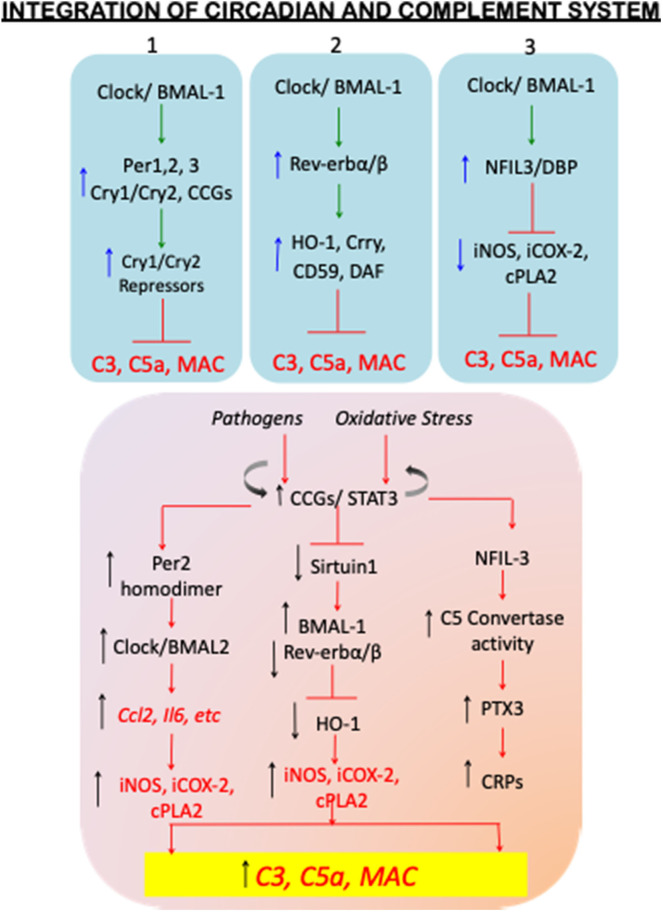
Homeostatic circadian and dysregulated circadian mechanisms with complement activation. The top panel (blue shade) of the flowchart shows the circadian regulatory proteins so far known to control complement activation under SCN homeostatic conditions. The blue upward arrows depict increase in the circadian regulatory protein levels, and the circadian-upregulated complement regulatory proteins CD59, Crry, and DAF, and antioxidant factors, such as hemoxygenase1 (HO-1) (top blue panels 1, 2, and 3), resulting in decreased levels of C3, C5a, and therefore inhibition of MAC formation. The blue downward arrows (top blue panel 3) show circadian-downregulated proinflammatory mediators iNOS, iCOX-2, cPLA2. The inhibitory signs are shown in red color. The dysregulated circadian mechanisms are schematically shown in the lower panel (orange shade). Immunopathogenesis and oxidative stress induced clock disruption results in constitutive activation of circadian controlled genes thereby increasing the levels of C3, C5a, and MAC. A few of the mechanism-based evidences include: dimerization of Per2 and its preferential binding to Clock-BMAL2 complex resulting in plausible increase in alternate complement activation; elevated BMAL1 and its hyper acetylation in the absence of sirtuin 1 and plausibly reduced Rev-erbα/β-HO-1-mediated anti-inflammatory cues; and increased NFIL-3-mediated C5 convertase activity and the plausible disrupted clock-activated Pentraxin-3 and C-reactive proteins; leading to uncontrolled complement activation. The details of these studies are discussed in the review text.

Cellular rhythms, including those within and external to the circadian clock are based on a transcriptional negative feedback loop. The two transcriptional activators CLOCK and BMAL1 form heterodimers that regulate downstream transcription of the cryptochrome (*Cry*) (Ripperger and Albrecht, 2012) and period (*Per*) (Gundel and Wegmann, 1987) genes. PER and CRY proteins accumulate in a cyclic manner and provide direct negative transcriptional feedback to CLOCK: BMAL1 (Figure 1 bottom; Figure 2; top-blue panel 1). Interestingly, *cry1/cry2* double knockout mice, have significantly increased C3, the classical pathway precursor of C3a anaphylatoxin, which results in activation of alternate pathway (Cao et al., 2017). *Cry1/Cry2*-deficient mice have a pronounced autoimmune phenotype, which includes elevated serum IgG and serum antinuclear antibodies. CRY-deficiency also leads to downregulation of the complement component C1q, which plays a key role in clearing immune complexes, and is considered preventative in the context of systemic lupus erythematosus (Gibbs et al., 2012), in which healthy cells and tissues are mistakenly attacked by the immune system (Mok and Lau, 2003). Similar to *Cry*-deficiency, deficiency of the complement inhibitor CRRY (homolog of MCP in humans) leads to significantly increased ischemic renal tubular injury. More importantly these mice also exhibit downregulation of other complement regulatory proteins, including decay accelerating factor (DAF) and protectin (CD59) (Renner et al., 2010). Given that most of the MCPs also exist as homodimers, these two independent studies therefore support the potential overarching regulatory effects of circadian clock machinery on complement immune system in the autoimmune lupus nephritis.

The second loop of CLOCK: BMAL1-dependent transcriptional activation involves Rev-erbα and Rev-erbβ that belong to the nuclear receptor family of proteins (also known as NR1D1/NR1D2). These proteins serve as transcriptional repressors of *Bmal1* transcription but also target a wide range of genomic loci (Schaefer et al., 2005; Zhang et al., 2015). While it has been shown that BMAL1 controls *Ccl2* expression in macrophages to maintain an anti-inflammatory state, stimulation of Rev-erbβ with agonist GSK4112 inhibits IL-6 production in LPS challenged mice in a *zeitgeber*-specific manner. This result was further supported in Rev-erbα KO mice, wherein circadian gating of *Il6* expression is lost, resulting in excessive inflammation closely associated with the innate immune system (Gibbs et al., 2012). Similar effects of GSK4112 have been demonstrated on Rev-erbβ-dependent inhibition of adipogenesis by heme oxygenase-1 (HO-1), which results in the expression of anti-inflammatory cytokines IL-10 and IL-1 receptor antagonist IL-RA (Kumar et al., 2010; Piantadosi et al., 2011). Importantly, synovial fluids of patients with rheumatoid arthritis contain excessive IgG and IgM autoantibody accumulation resulting in complement activation, and monocytic and neutrophil infiltration in the synovial joints (Massberg et al., 2007). This causes tissue destruction via MAC (Cao et al., 2017) and exacerbating effector cell inflammation (Perez-Aso et al., 2013; Sadik et al., 2018). Although anti-inflammatory components, such as HO-1 are also induced in the synovial joints, the levels of HO-1 are shown to decline with the advanced immunopathogenesis of RA (Kitamura et al., 2011; May et al., 2018), which is presumably moderated by the RA-driven proinflammatory circadian responses (Lee et al., 2017). Thus, the second regulatory loop of the core transcriptional circadian clock proteins also plays an important role in immune response and complement signaling (Figure 2-top blue panel 2; bottom orange panel).

Additional proteins contributing to cellular rhythmicity on a global scale involves CLOCK:BMAL1-activated D-box binding protein (DBP) and nuclear factor IL3 (NFIL3), which control transcriptional activation of D-box- containing genes including *Per* genes via enhanced binding of NFkB on the *Per2* promoter region (Yamajuku et al., 2011; Hong et al., 2018). Owing to the fact that NFIL3 stimulates NFkB gene expression, NFIL3 indirectly activates cellular proliferation of the hematopoietic population in the bone marrow. While NFIL-3 is reported to enhance *Per2* expression in isolated and cultured synovial cells when stimulated by TNFα, during progression rheumatoid arthritis (Yoshida et al., 2013), it has also been shown to act as a transcriptional repressor reducing inflammation in dexamethasone induced-ID-13 fibroblast-like synoviocytes by transcriptionally repressing inflammatory genes inducible nitric oxide synthase (Gustavsson et al., 1995) (*inducible nitric oxide synthase*), *COX-2* (*Cyclo-oxygenase-2*), and *cPLA2* (*cytoplasmic phospholipase A2*) (Wallace et al., 1997) (Figure 2-bottom orange panel). In addition, there is significantly increased expression of a both circadian and complement genes, such as *Dbp, Per2, Npas2* (homologto *Clock*), *A*rntl (BMAL1), and the proven osteoarthritis markers, C1q, C3, and C5aR1, found in the joint tissues obtained from experimental temporomandibular joint-osteoarthritis (TMJ-OA) rat models (He et al., 2018).

In systemic influenza-like viral infections, or upper respiratory tract infections, Fananapazir et al. (1977), reported heightened levels of serum IgG, C3 and fibrin-degradation products that were also measurable in urinary samples of afflicted patients. Similar activation of fibrinolytic cascade along with the alternate complement cascade has been demonstrated by Borkowska et al. (2014), with NFIL3-mediated increase in C5 convertase activity generating C5a during mobilization of HSPCs from the bone marrow to the peripheral blood. These results are similar to a previous study, showing circadian-regulated changes might be involved in conditions of stress, infections, tissue damage, and strenuous exercise. Therefore, complement activation and the C5a/C5aR1 axis is directly associated with circadian controlled gene stimuli (Borkowska et al., 2014). In patients with paroxysmal nocturnal hemoglobinuria (PNH), autologous complement activation has been demonstrated as a result of CD59 and DAF deficiency, inhibiting MAC formation resulting in hemolysis (Young et al., 2009; Parker, 2016). *C5aR1* and complement factor P (*CFP*) also known as *Properdin*, and circadian associated genes were also shown to exacerbate IL-5 and IL-13, the type 2 inflammatory cytokines and inflammasome pathway activation involving eosinophils in allergic rhinitis patients (Leaker et al., 2017). Reciprocal to upregulation of *C5aR1* and *CFP*, there was a downregulation of *C1qA* and *C1R* expression, suggesting exacerbated mast cell activation, which leads to eosinophilic type 2 inflammation and Th17 allergic response (Schmudde et al., 2013).

Consistent with these studies, nasal mucosal samples of these PNH patients also show upregulated *Bmal1*, Retinoic acid-related receptors, *Rora* and *Rorc*, with downregulation of repressive genes including *Per1-3*, and *Rev-erb*α. These results also correlated with studies on mouse airway inflammation which shows that cigarette smoking in C57B6/J mice results in decreased levels of *Per1, Rev-erb*α. Interestingly BMAL1 appears to be hyper-acetylated because of the absence of Sitruin1 (*Sirt1*) (Hwang et al., 2014). These data independently corroborate those showing the increased C5a/C5aR1 signaling due to elevated BMAL1 in autoimmune pathogenesis (discussed above), and that a reduction in Sirtuin1 function may correspond to immunopathogenesis (Gerhart-Hines et al., 2011; Hernandez et al., 2017; Tamura et al., 2017) (Figure 2-bottom orange panel).

## Complement and Circadian Systems in Metabolic Diseases

Epidemiological studies looking at individuals with irregular sleep patterns, such as those working irregular hours and in day/night shift changed-schedules have demonstrated an increased occurrence of autoimmune diseases, as discussed above, along with metabolic diseases type 2 diabetes, liver disease, and inflammatory bowel disease, compared to individuals working normal shifts (Lundasen et al., 2006; Marcheva et al., 2010; Sobolewska-Wlodarczyk et al., 2016; Mindikoglu et al., 2017; Gombert et al., 2019; Shoar et al., 2019). While impaired entrainment in the inflammatory tissues can be a secondary consequence of disrupted SCN rhythmicity with chronic metabolic dysfunction and lifestyle, intestinal microbiota also contributes to proinflammatory innate immune cues generated from the antigen presenting cells, such as dendritic cells and macrophages, through a TLR2/MyD88 pathway resulting in B and T lymphocytes proliferation and activation. Interestingly, samples of brain and blood isolated from mice with Rett syndrome (caused by specific mutations in *Mecp2-methyl CpG protein2*, a gene that encodes MECP2 protein important in mature nerve cell functioning), showed shared mechanisms of circadian controlled genes with complement activation lipid metabolism and stress response (Yang et al., 2017). Elevated complement cascade-associated genes, Serpin1, and *Ube2V1* of the ubiquitination system, suggest a therapeutic approach that targets ubiquitous-altered mechanisms for better protection of stress-related progression of the disease (Sanfeliu et al., 2019). In regard to reproductive physiology, these three key pathways of inflammation, the proteasome, complement, and coagulation cascades were also shown to interact with circadian regulated genes in the endometrial biopsy samples isolated from female patients having repeated implantation failure (Bastu et al., 2019). Complement regulatory protein, MCP also known as CD46, was significantly upregulated in these patients, likely due to increased C5a/C5aR1 axis. This correlated with the downregulation of *Clock* gene expression and that of succinate dehydrogenase C (SDH-C), of the citric acid cycle. Thus, the inflammation associated with hypoxia and altered metabolite partitioning resulted in the failure of the blastocyst implantation. Importantly, angiotensin I (*AGT*), and its endopeptidase neprilysin, *MME*, were also upregulated in these patients, suggesting hypertension in the uterine vascular circuits may also be contributing to the failure of blastocyst implantation (Bastu et al., 2019).

Rhythmicity of metabolism in some cell types appears to also be necessary for appropriate immune response. One study aimed at understanding immunological phagocytosis in human retinal pigment epithelium, revealed that phagocytosis of beads containing microbial pathogens *Staphylococcus aureus*, and *Escherichia coli*, was diurnal and coincident with IFN-gamma-induced neopterin production (Irschick et al., 2004, 2009). However, chronic inflammatory autoimmune diseases, such as macular degeneration do activate the alternate complement pathway (Lechner et al., 2016; Pujol-Lereis et al., 2016). In macular degeneration, mannose binding receptor levels are substantially elevated, which is suggestive of an uncontrolled alternate complement pathway (Wilt and McLaughlin, 1999; Wilt et al., 1999). Interestingly, though no direct links between the clock and complement in this system have been explored to date, BMAL1-mediated dysregulation of the *claudin-5* (gene encoding a tight junction protein expressed in the inner blood-retina barrier) can under nutrient stress conditions cause retinal pigment epithelium cell atrophy in a non-human primate model system (Hudson et al., 2019). Angiography in both human and non-human primates reveals higher retinal vascular permeability at the evening and lower levels in the morning.

So, to what extent do these changes in circadian gene expression actually affect transplant-mediated immune response and survival of transplant tissue? The answer is not entirely clear. However, studies in circadian mutant models do reveal the importance of the clock system in transplant response. A remarkable study using *Bmal1* or *Per2/3* knockout mice revealed that while grafting WT blood vessels into circadian mutant mice produced minimal to no phenotype, whereas grafting aortic vessels from circadian mutant mice into a WT host resulted in severe arteriosclerotic disease (Cheng et al., 2011). This was associated with increased macrophage receptor CD68, and elevated T cell receptors, both markers of transplant rejection. This also correlated with increased leukocyte infiltration. Thus, transplant response is impinged upon by the circadian clock system. Additional circadian regulated factors can also affect transplant response. Circadian-regulated Pentraxin 3, PTX-3, is activated by complement in metabolic tissue transplantation patients resulting in post-transplant complications (Castellano et al., 2010; Csincsi et al., 2015; Roy et al., 2017). Interestingly, Pentranxin-3 is highly elevated in night-shift workers (Pavanello et al., 2017) coincident with increased total C-reactive protein levels, body mass index and cardiovascular diseases and hypertension (Figure 2-bottom orange panel). In addition to complement activation, presence of systemic proteases and thrombin activation under chronic oxidative stress conditions also enhance C5a levels (Krisinger et al., 2012). Castellano et al. have demonstrated that C5a/C5aR1 signaling results in acceleration of renal tubular cell senescence via Wnt/β-catenin pathway (Castellano et al., 2019), which is not surprising because activated alternate pathway is addressed in glomerulonephropathies, and renal transplantation associated complications that arise from the activated complement cascade (Nauser et al., 2017). Thus, Figure 2 depicts some of the plausible pathological changes that alter key circadian pathways and lead to uncontrolled activation of complement cascade.

## Potential Therapeutic Interventions and Future Directions

**Figure 3:**
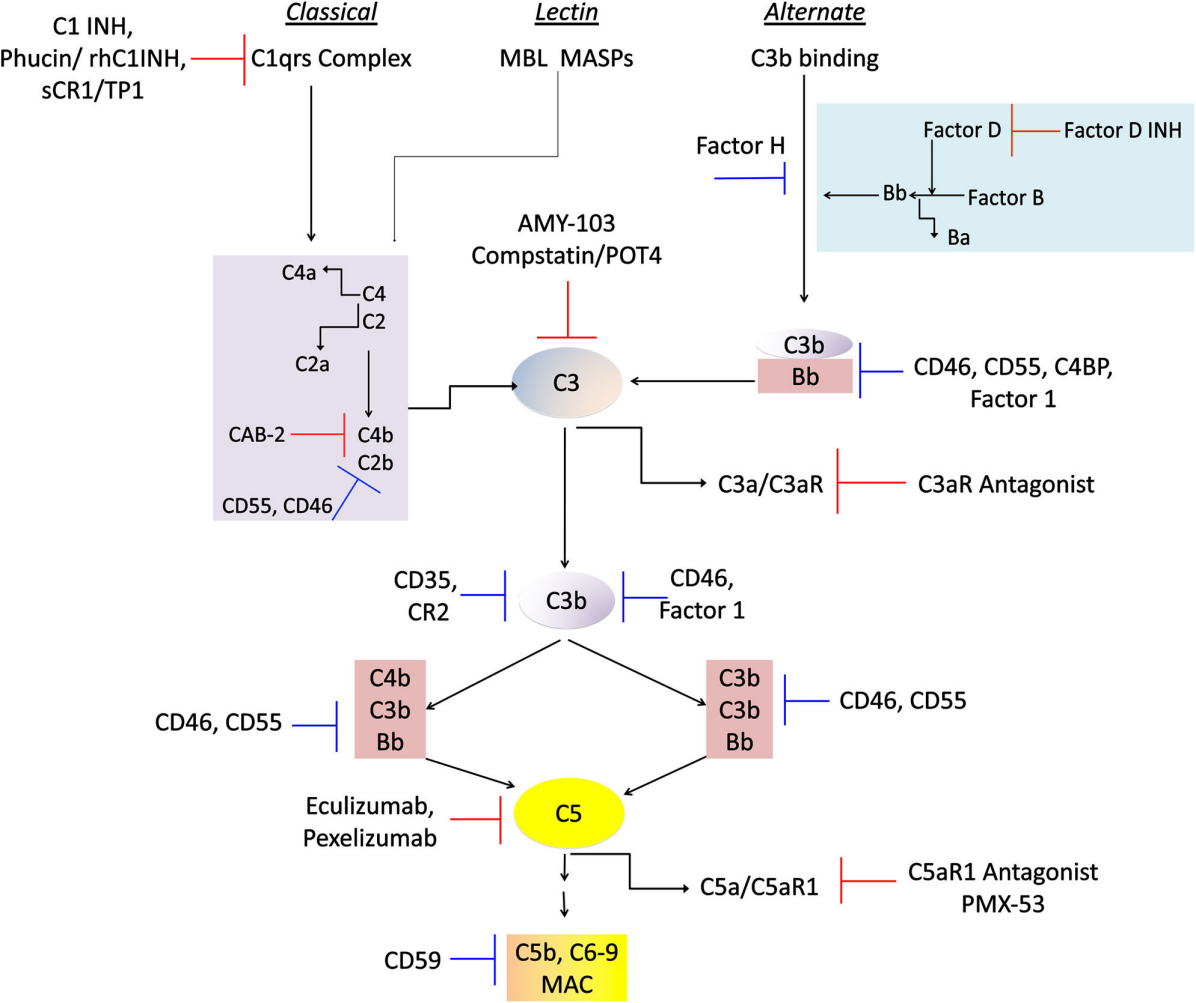
Physiological regulators and therapeutic drugs in complement control. A flowchart showing the physiological regulatory proteins and clinical interventions so far known to regulate chronic inflammation in afflicted patients. The targeted activities of these molecules include regulation at all the three complement cascades that activate the C3 component that leads to generation of potent anaphylatoxins C3a, and C5a, and the MAC formation. The blue inhibition mark denotes physiological proteins involved in regulation of these pathways, and the red blocking sign denotes pharmacological inhibitors being employed in clinical trials and/or therapeutic approaches. The details of the companies and their phases of clinical trials are described in the review text.

C3 complement, with the reference values of complement C3 ranging 900–1,800 mg/L in human serum, lies at the center of classical and alternate pathways, in health, pathogenic assaults, and chronic inflammatory diseases. Therefore, it is imperative that robust inhibitory mechanisms are in place for regulating alternate pathway activation as well as inactivation of C3a and C5a, that are typically generated in a constitutive manner. Such inhibition is essential for keeping the levels of C3a and C5a at physiological levels. Interestingly, cancer cells produce high levels of complement inhibitors, such as factor H, CD55 and CD46, and therefore therapeutics have to be carefully chosen for disease specific targeting (Markiewski et al., 2008). However, several therapeutics related to complement signaling have been tested and either have been or are currently in clinical trials (Ricklin and Lambris, 2007; Hajishengallis and Lambris, 2013). For example, as depicted in Figure 3, Factor H targeting by factor D inhibitor, Lampalizumab (Roche AG, Basel, Switzerland) is in clinical trials for age-related macular degeneration (AMD) and orphan renal disease. CD55 (DAF) and CD46 (MCP) inhibitors, are presently in clinical trials for coronary artery bypass grafting (CABG) (Ricklin and Lambris, 2007; Kulkarni and Afshar-Kharghan, 2008). More importantly C5 activation and its peptide C5a signaling are in the FDA-approved clinical interventions to this date: PMX-53™ (PepTech, MA) is prescribed for rheumatoid arthritis and psoriasis patients, and Eculizumab™ (Alexion, MA) is prescribed for paroxysmal nocturnal hemonglobinuria. Pexelizumab (Alexion, MA), a monoclonal antibody against C5 is under phase 3 clinical trials for Acute myocardial infarction and CABG (Mathew et al., 2004; Kulkarni and Afshar-Kharghan, 2008). Drugs affecting complement 3 signaling are also being studied. Complement 3 is clinically targeted by FDA-approved AMY-103 (Amyndas, PA) in transplant patients, and Compstatin/POT4 (Potentia Pharmaceuticals) is in phase clinical trials for treating AMD. Complement receptor 1 inhibitor sCR1/TP10 is under phase 2 clinical trials for CABG (Ricklin and Lambris, 2007). Two other potential therapeutics of C1 targeting, namely Phucin/rhC1INH (Pharmin Group, N.V., Leiden, The Netherlands) and C1-INH (Cetor, BerinertP, Leve Pharma) are both in phase 3 clinical trials (Ricklin and Lambris, 2007). Thus, there is tremendous opportunity for chronotherapy and it is likely that the circadian properties of complement signaling, and response will need to be taken into account for optimal efficacy of some of these compounds. Though the relationship between the circadian clock and complement signaling is still being actively envisaged, sufficient evidence suggests that like most cellular processes, the complement system is highly rhythmic, and yet another component of human physiology that can be compromised by forms of circadian disruption.

## Author Contributions

PS conceived the idea and wrote the original manuscript. BF reviewed the manuscript and provided technical assistance. KE-M contributed to the manuscript drafting and provided support on circadian studies. RW provided overall guidance, provided support on complement immunity studies, edited the manuscript, and approved the submitted version.

## Conflict of Interest

The authors declare that the research was conducted in the absence of any commercial or financial relationships that could be construed as a potential conflict of interest.
